# Tunable resistivity of correlated VO_2_(A) and VO_2_(B) via tungsten doping

**DOI:** 10.1038/s41598-020-66439-2

**Published:** 2020-06-16

**Authors:** Songhee Choi, Gihyeon Ahn, Soon Jae Moon, Shinbuhm Lee

**Affiliations:** 1 Department of Emerging Materials Science, Daegu-Gyeongbuk Institute of Science and Technology, Daegu, 42988 Republic of Korea; 2 Department of Physics, Hanyang University, Seoul, 04763 Republic of Korea

**Keywords:** Condensed-matter physics, Materials for devices

## Abstract

Applications of correlated vanadium dioxides VO_2_(A) and VO_2_(B) in electrical devices are limited due to the lack of effective methods for tuning their fundamental properties. We find that the resistivity of VO_2_(A) and VO_2_(B) is widely tunable by doping them with tungsten ions. When *x* < 0.1 in V_1*−x*_W_*x*_O_2_(A), the resistivity decreases drastically by four orders of magnitude with increasing *x*, while that of V_1*−x*_W_*x*_O_2_(B) shows the opposite behaviour. Using spectroscopic ellipsometry and X-ray photoemission spectroscopy, we propose that correlation effects are modulated by either chemical-strain-induced redistribution of V−V distances or electron-doping-induced band filling in V_1*−x*_W_*x*_O_2_(A), while electron scattering induced by disorder plays a more dominant role in V_1*−x*_W_*x*_O_2_(B). The tunable resistivity makes correlated VO_2_(A) and VO_2_(B) appealing for next-generation electronic devices.

## Introduction

Since the level of correlation effects between neighbouring electrons in the outermost 3*d* orbitals of vanadium ions differs depending on the crystal structure, the polymorphs of vanadium dioxide (VO_2_) show a wide range of electrical properties, acting as an insulator in monoclinic VO_2_(M1) and tetragonal VO_2_(A), a semiconductor in monoclinic VO_2_(B), and a conductor in tetragonal VO_2_(R)^1–3^. Therefore, VO_2_ polymorphs have been extensively studied for a range of interesting applications. VO_2_(M1) and VO_2_(R) have attracted wide interest for electronic devices since they show heat-, light-, electric field-, and chemical-induced reversible metal-insulator transitions near room temperature (*T*_MI_ = 340 K in bulk)^4–7^. On the other hand, VO_2_(A) and VO_2_(B) have been mainly used for energy applications, including redox-flow batteries, ion batteries, solid oxide fuel cells, hydrogen storage devices, and catalysts^8^. Compared to the many studies on electronic devices using VO_2_(M1) and VO_2_(R), however, the scarcity of published methods to tune the electrical properties of correlated VO_2_(A) and VO_2_(B) has limited their potential applications in electrical devices.

Several methods have been suggested for tailoring the correlation effects of VO_2_(M1) since broad tunability of electrical properties is valuable for electrical devices. In addition to metallization induced by pressure application^9^, hydrogen doping^10,11^, or ionic liquid gating^12,13^, cation doping has been widely used as a method with high efficacy. Chromium doping can result in transition of the dimerization of V−V chains in the VO_2_(M1) phase into partial dimerization in the VO_2_(M2) phase^14^. Substituting a small amount of tungsten for vanadium in VO_2_(M1) causes notable changes in its electrical property^15^. For convenience, V_1*−x*_W_*x*_O_2_ will be used herein to denote VO_2_ with tungsten doping of *x* × 100%. As *x* increases, the resistivity of V_1*−x*_W_*x*_O_2_ films decreases by several orders of magnitude, and *T*_MI_ also rapidly decreases at a rate of *dT*_MI_/*dx* = 2,100–2,800 K. For 0.08 < *x* < 0.09, the V_1*−x*_W_*x*_O_2_ epitaxial films have a metallic ground state for a wide temperature range of 50–400 K. When *x* is increased beyond this range, V_1*−x*_W_*x*_O_2_ reenters its insulating state.

Exploring tuning knobs of the electrical properties of VO_2_(A) and VO_2_(B) would not only enable their usage in electrical devices but also promote rich functionalities for energy devices. Motivated by research on correlated VO_2_(M1), here, we investigate the effects of tungsten doping on the resistivity of correlated VO_2_(A) and VO_2_(B). We organize this paper as follows: first, we describe how, as the tungsten concentration increases, chemical strain increases the lattice parameters of both VO_2_(A) and VO_2_(B). Next, we find that tungsten doping is effective for tuning the resistivity of correlated VO_2_(A) and VO_2_(B) over a broad range. For *x* < 0.1–0.15, VO_2_(A), an insulator in the pure phase, exhibits a monotonic decrease in resistivity of four orders of magnitude with increasing *x*. VO_2_(B), a semiconductor in the pure phase, shows a monotonic increase in the resistivity of two orders of magnitude. Finally, to understand these opposite dependences, we explore the systematic evolution of the electronic structures and vanadium oxidation states by performing spectroscopic ellipsometry and X-ray photoemission spectroscopy (XPS), respectively.

### Chemical tensile strain in VO_2_(A) and VO_2_(B) induced by tungsten doping

**Figure 1:**
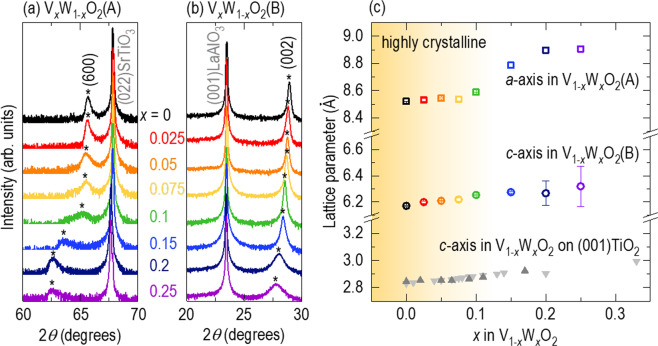
X-ray diffraction (XRD) *θ*‒2*θ* scans for (**a**) V_1*−x*_W_*x*_O_2_(A) (*x* ≤ 0.25) epitaxial films grown on (011)-oriented SrTiO_3_ and (**b**) V_1*−x*_W_*x*_O_2_(B) epitaxial films grown on (001)-oriented LaAlO_3_. The diffraction peaks of the films, highlighted by asterisks (*), are preserved for *x* < *x*_c_ ≈ 0.1–0.15, but these intensities become weaker for *x* > *x*_c_ due to film deterioration. With increasing tungsten concentration, V_1*−x*_W_*x*_O_2_(A) and V_1*−x*_W_*x*_O_2_(B) show a shift in their diffraction peaks towards lower 2*θ* values. (**c**) Tungsten concentration dependence of the lattice parameters of V_1‒*x*_W_*x*_O_2_(A) and V_1‒*x*_W_*x*_O_2_(B). We compare our results to the lattice parameters of V_1‒*x*_W_*x*_O_2_ epitaxial films grown on (001)-oriented TiO_2_ (light grey^15^ and grey^20^ triangles). The lattice parameters show monotonic increases with increasing tungsten concentration. The region in graded yellow indicates preservation of the crystal structures when *x* < *x*_c_.

Using pulsed laser epitaxy, we grew (100)-oriented V_1*−x*_W_*x*_O_2_(A) and (001)-oriented V_1*−x*_W_*x*_O_2_(B) epitaxial films on (011)SrTiO_3_ and (001)LaAlO_3_, respectively, for *x* from 0 to 0.25. We provided details of the deposition conditions in our previous reports^2,3,16–18^ as well as in the Methods section. Figure 1a,b show the X-ray diffraction (XRD) *θ* − 2*θ* scans of V_1*−x*_W_*x*_O_2_(A) and V_1*−x*_W_*x*_O_2_(B), respectively (see Fig. S1 for XRD *θ* − 2*θ* scans in a wider 2*θ* range). We clearly observed (600)VO_2_(A) diffraction peaks for *x* < 0.1 and (002)VO_2_(B) diffraction peaks for *x* < 0.15, indicating preservation of the crystal structures for *x* < *x*_c_ ≈ 0.1–0.15. However, for *x* > *x*_c_, these XRD intensities significantly decreased, and some peaks disappeared, indicating that heavy doping of tungsten could lower the quality of our epitaxial VO_2_(A) and VO_2_(B) films [we ruled out possible effects of film thickness on the reduction of the XRD intensities since the X-ray reflectivity results (Fig. S2) revealed an almost invariant thickness of ~100 nm with tungsten doping.]. This observation is different from the seemingly preserved crystallinity up to *x* = 0.33 in V_1*−x*_W_*x*_O_2_ epitaxial films grown on (001)-oriented TiO_2_^15^, which might be due to a strong strain effect between isostructural VO_2_(R) and TiO_2_. Hereafter, we will mainly focus on the resistivity of V_1−*x*_W_*x*_O_2_ (*x* < *x*_c_) to avoid unwanted effects from film deterioration.

Substitution with tungsten ions, which are larger (0.60 Å)^19^ than vanadium ions (0.58 Å), causes chemical tensile strain in V_1*−x*_W_*x*_O_2_(A) and V_1*−x*_W_*x*_O_2_(B) epitaxial films. As shown by the XRD *θ* − 2*θ* scans, the (600)VO_2_(A) diffraction peak gradually shifts to a lower 2*θ* angle with increasing tungsten concentration (Fig. 1a), indicating an increase in the *a*-axis lattice parameter. We observe a similar behaviour for V_1*−x*_W_*x*_O_2_(B) films. The observation of the (002)VO_2_(B) diffraction peak at a lower 2*θ* angle with tungsten doping (Fig. 1b) indicates an increase in the *c*-axis lattice parameter. As shown in Fig. 1c, the *a*- and *c*-axis lattice parameters of V_1*−x*_W_*x*_O_2_(A) and V_1*−x*_W_*x*_O_2_(B) increase by 4.8% from 8.52 to 8.91 Å and by 2.4% from 6.17 to 6.32 Å, respectively, for *x* = 0–0.25. This chemical tensile strain is similar to the increase in the *c*-axis lattice parameter by 5.7% from 2.83 to 2.99Å for *x* = 0–0.33 in V_1*−x*_W_*x*_O_2_ epitaxial films grown on (001) TiO_2_ (for convenience of this calculation, we assumed that V_1*−x*_W_*x*_O_2_ epitaxial films have a tetragonal structure on (001)TiO_2,_ although R, M1, and the intermediate phases could coexist in one film, as indicated by a broadened *T*_MI_)^15,20^.

### Tunable resistivity of VO_2_(A) and VO_2_(B) with tungsten doping

**Figure 2:**
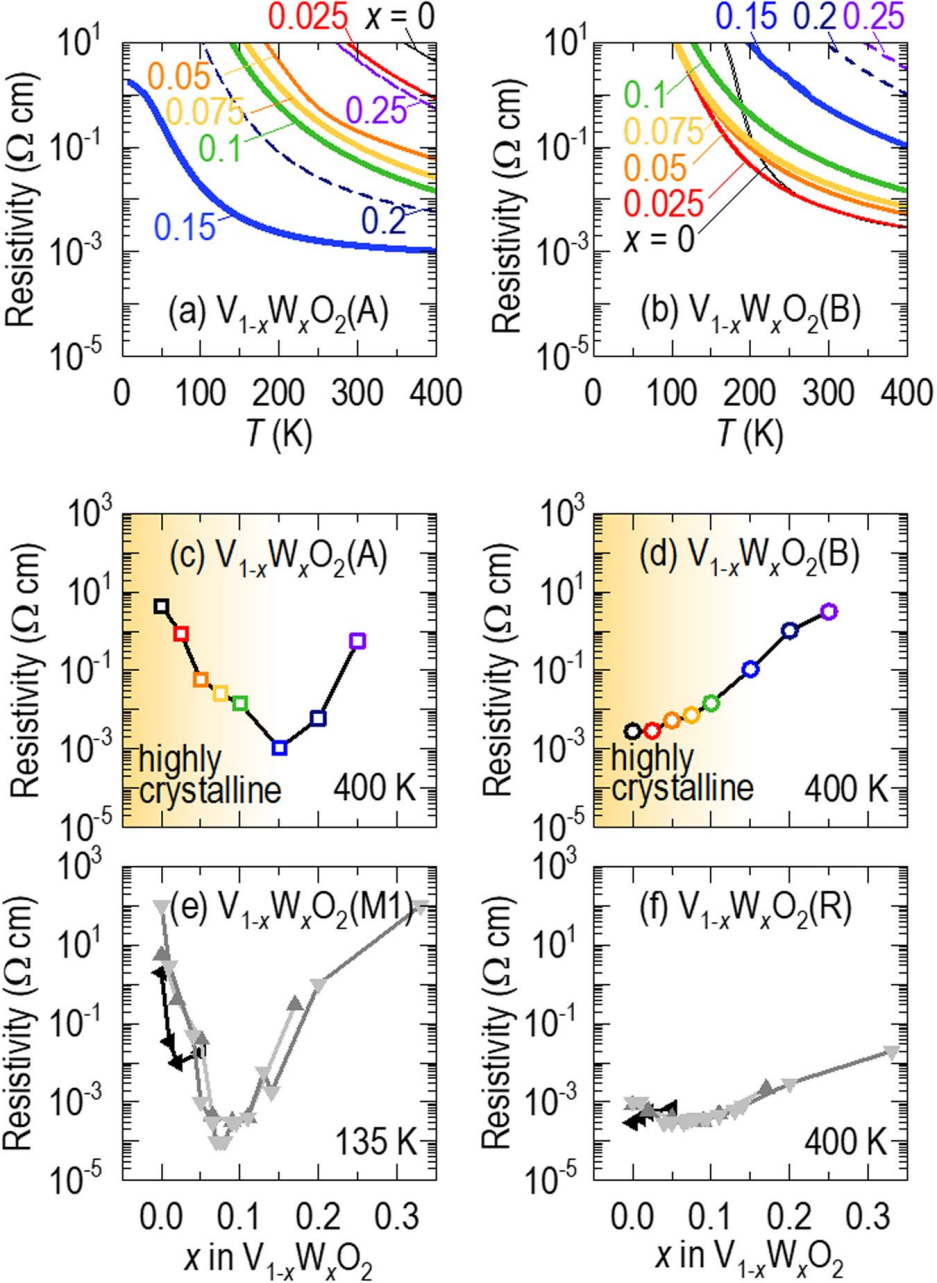
Tungsten concentration dependence of the resistivity-temperature curves of (**a)** V_1‒*x*_W_*x*_O_2_(A) and (**b**) V_1‒*x*_W_*x*_O_2_(B). The resistivity of the insulating VO_2_(A) phase decreases as the tungsten concentration increases up to *x*_c_ (solid lines) but increases for *x* > *x*_c_ (dashed lines). By contrast, the resistivity of the semimetallic VO_2_(B) phase increases with increasing tungsten concentration for all *x* values. Comparison of the tungsten concentration dependences of the resistivity between **(c**) V_1‒*x*_W_*x*_O_2_(A) at 400 K, (**d**) V_1‒*x*_W_*x*_O_2_(B) at 400 K, (**e**) V_1‒*x*_W_*x*_O_2_(M1) at 135 K, and (**f**) V_1‒*x*_W_*x*_O_2_(R) at 400 K. We plotted data for V_1‒*x*_W_*x*_O_2_(M1) and V_1‒*x*_W_*x*_O_2_(R) from the literature (light grey^15^, grey^20^, and black^22^ triangles). For *x* < *x*_c_, the resistivities of insulating (A and M1) and metallic (B and R) materials decrease and increase, respectively. For *x* > *x*_c_, the resistivities of all films increase. The region in graded yellow for *x* < *x*_c_ indicates that the crystal structures are preserved.

With increasing tungsten concentration (*x* < *x*_c_), the resistivity of VO_2_(A) decreased, while that of VO_2_(B) increased. Figure 2a shows the temperature dependence of the resistivity of V_1*−x*_W_*x*_O_2_(A) for *x* = 0–0.25. The resistivity of pure VO_2_(A) was very high, i.e., 4.37 Ω cm at 400 K, and increased with decreasing temperature. Such insulating behaviour is attributed to correlation-induced bandgap opening between unoccupied and occupied *t*_2g_ orbitals^1^. It should be noted that the resistivity of V_1*−x*_W_*x*_O_2_(A) decreased with *x* (<*x*_c_, solid lines). The V_1*−x*_W_*x*_O_2_(A) epitaxial film for *x* = 0.15 showed a smaller resistivity (by three orders of magnitude) of 0.001 Ω cm at 400 K than that of pure VO_2_(A). This small resistivity indicated that the film was on the verge of metallicity, in terms of the Mott-Ioffe-Regel limit^21^ (i.e., the material is regarded as a metal when the resistivity is smaller than 0.001 Ω cm.). The resistivity of V_1*−x*_W_*x*_O_2_(A) increased for *x* > *x*_c_ (dashed lines), probably due to film deterioration. As shown in Fig. 2b, the resistivity of pure VO_2_(B) also increased with decreasing temperature, indicating insulating behaviour. However, its resistivity was close to the Mott-Ioffe-Regel limit^21^ at 400 K and significantly smaller (by three orders of magnitude) than that of pure VO_2_(A). Such a low resistivity in pure VO_2_(B) is ascribed to thermal electron jumping across the very narrow bandgap (<25 meV) near room temperature^1^. The resistivity of V_1*−x*_W_*x*_O_2_(B) increased with increasing *x* by two orders of magnitude. Moreover, the resistivity increased more for *x* > *x*_c_, as observed in V_1*−x*_W_*x*_O_2_(A).

We noted interesting features of the tungsten doping effects on the resistivity of V_1*−x*_W_*x*_O_2_(A) and V_1*−x*_W_*x*_O_2_(B) (*x* < *x*_c_). The dependences were opposite: the resistivity of V_1*−x*_W_*x*_O_2_(A) decreased, i.e., 4.37 → 0.01 Ω cm at 400 K for *x* = 0 → 0.1, while V_1*−x*_W_*x*_O_2_(B) showed increasing resistivity, i.e., 0.003 → 0.01 Ω cm. It is surprising that the resistivities of VO_2_(A) and VO_2_(B) changed to such an extent, although we doped a relatively small amount (*x* < 0.1) of tungsten ions. Therefore, our work indicates that tungsten doping is promising for tuning the resistivity of VO_2_(A) and VO_2_(B). This extensive tunability is expected to provide many opportunities to realize both electronic and energy devices using correlated VO_2_(A) and VO_2_(B).

To obtain more information about these opposing and large dependences, we compared the tungsten-doping dependences of the resistivity in V_1*−x*_W_*x*_O_2_(A) and V_1*−x*_W_*x*_O_2_(B) to those in previous reports on V_1*−x*_W_*x*_O_2_(M1) and V_1*−x*_W_*x*_O_2_(R). It was simple to evaluate the resistivities of our V_1*−x*_W_*x*_O_2_(A) and V_1*−x*_W_*x*_O_2_(B) epitaxial films since, across a wide temperature range, they do not show any phase transitions. However, when we plotted the resistivities of V_1*−x*_W_*x*_O_2_(M1) and V_1*−x*_W_*x*_O_2_(R), we had to pay attention to the different resistivities of the M1 and R phases due to the *T*_MI_ variation induced by the tungsten doping. Figure 2c–f show the 400 K resistivities of V_1*−x*_W_*x*_O_2_(A) and V_1*−x*_W_*x*_O_2_(B), the 135 K resistivity of V_1*−x*_W_*x*_O_2_(M1)^15,20,22^, and the 400 K resistivity of V_1*−x*_W_*x*_O_2_(R)^15,20,22^. We note three features of the tungsten doping effect across the polymorphs. (1) For light doping (*x* < *x*_c_), at which the crystal structures are well preserved (highlighted in yellow), the resistivities of insulating VO_2_(A) and VO_2_(M1) decrease by 3–4 orders of magnitude compared to those in the pure phases, while semiconducting VO_2_(B) and metallic VO_2_(R) exhibit increases in the resistivity by 1–2 orders of magnitude compared to those in the pure phases. (2) It is quite surprising that the resistivities of V_1*−x*_W_*x*_O_2_(A) and V_1*−x*_W_*x*_O_2_(M1) can be smaller than those of V_1*−x*_W_*x*_O_2_(B) and V_1*−x*_W_*x*_O_2_(R) when doped with certain amounts of tungsten (e.g., *x* ≈ *x*_c_ in this work). (3) For heavy doping (*x* > *x*_c_), all phases show increased resistivity. We suggest that the increases seen in V_1*−x*_W_*x*_O_2_(A) and V_1*−x*_W_*x*_O_2_(B) can be attributed to deterioration of the films because we observed suppression of diffraction peaks in the *θ*−2*θ* XRD scans (Fig. 1a,b). The similar dependences between VO_2_(A) and VO_2_(M1) and between VO_2_(B) and VO_2_(R) suggest that the proposed mechanisms underlying the tungsten doping effects in VO_2_(M1) and VO_2_(R) may apply to VO_2_(A) and VO_2_(B), respectively.

### Electronic structures of tungsten-doped VO_2_(A) and VO_2_(B)

**Figure 3:**
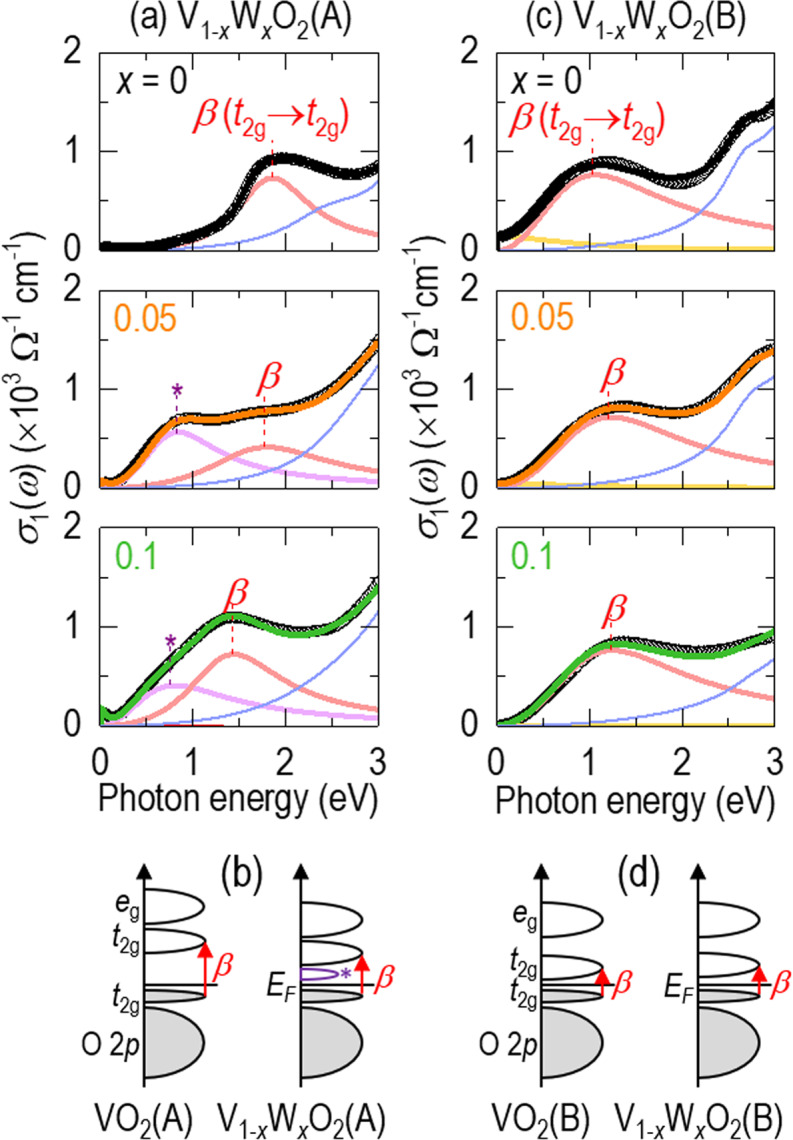
Evolution of the electronic structures of tungsten-doped VO_2_(A) and VO_2_(B). (**a**) Optical conductivity, *σ*_1_(*ω*), of V_1*−x*_W_*x*_O_2_(A) (*x* = 0, 0.05, 0.1). Open circles represent experimentally measured *σ*_1_(*ω*), and solid lines represent Lorentz oscillators. Due to optical transitions from occupied *t*_2g_ to unoccupied *t*_2g_ levels, the *β*-peak moves slightly towards lower photon energy for higher *x*. A new in-gap state (asterisk) appears near 0.8 eV. (**b**) Schematics of the electronic bandstructures of pure VO_2_(A) and V_1*−x*_W_*x*_O_2_(A). (**c**) *σ*_1_(*ω*) of V_1*−x*_W_*x*_O_2_(B) (*x* = 0, 0.05, 0.1). For higher *x*, the *β*-peak moves slightly towards higher photon energy, and the Drude response (yellow line) is slightly suppressed. **(d**) Electronic bandstructure schematics of pure VO_2_(B) and V_1*−x*_W_*x*_O_2_(B).

To better understand the opposing dependences on tungsten doping, we investigated the electronic structures of V_1*−x*_W_*x*_O_2_(A) and V_1*−x*_W_*x*_O_2_(B) epitaxial films for various *x* values. Figure 3a shows the optical conductivity *σ*_1_(*ω*) of V_1*−x*_W_*x*_O_2_(A) (*x* = 0, 0.05, 0.1) as a function of photon energy. Pure VO_2_(A) (first row) exhibited opening of a correlation-induced bandgap^1^. For *x* = 0.05 (second row), a spectral weight distinctly appeared near 0.8 eV. For *x* = 0.1 (third row), the bandgap might be narrower than 25 meV at room temperature (Figs. S3 and S4), so the electrons in the occupied *t*_2g_ orbital could thermally jump into the unoccupied *t*_2g_ orbitals even at room temperature, consistent with the low resistivity shown in Fig. 2a. To examine the variation in optical spectra with *x* in more detail, we evaluated *σ*_1_(*ω*) using Lorentz oscillators, ${{\rm{\sigma }}}_{1}(\omega )=\frac{{e}^{2}}{{m}^{\ast }}\frac{{N}_{D}{\gamma }_{D}}{{\omega }^{2}+{\gamma }_{D}^{2}}+\frac{{e}^{2}}{{m}^{\ast }}\sum _{j}\frac{{N}_{j}{\gamma }_{j}{\omega }^{2}}{{({\omega }_{j}^{2}-{\omega }^{2})}^{2}+{\gamma }_{j}^{2}{\omega }^{2}}$, where m*, *γ*_*j*_, and *ω*_*j*_ are the effective mass, damping coefficient, and angular frequency of the *j*^th^ resonance line, respectively^23^. The first and second terms in *σ*_1_(*ω*) represent the metallic Drude response and interband transitions, respectively. The *β*-peak represents an interband transition from occupied *t*_2g_ to unoccupied *t*_2g_ orbitals and shifts to a lower photon energy with increasing *x*. Additionally, a new peak (asterisk) appeared near 0.8 eV, representing the creation of an in-gap state between the occupied *t*_2g_ and unoccupied *t*_2g_ orbitals. The spectroscopic findings are consistent with the decrease in resistivity in V_1*−x*_W_*x*_O_2_(A). It should be noted that the spectroscopic results for V_1*−x*_W_*x*_O_2_(A) are similar to observations for V_1*−x*_W_*x*_O_2_(M1)^24^. Figure 3b shows proposed changes in the electronic structures of VO_2_(A) with tungsten doping, i.e., a shift of the unoccupied *t*_2g_ orbital towards the Fermi level and the appearance of a new in-gap state just above the Fermi level.

Different from V_1*−x*_W_*x*_O_2_(A), the electronic structure of V_1*−x*_W_*x*_O_2_(B) did not show any obvious changes. Figure 3c shows the *σ*_1_(*ω*) of V_1*−x*_W_*x*_O_2_(B) (*x* = 0, 0.05, 0.1) as a function of photon energy. Pure VO_2_(B) in the first row exhibited a non-negligible spectral weight near zero photon energy^1^, consistent with its low resistivity near room temperature shown in Fig. 2b. With increasing tungsten concentration, the *β-*peak moved very slightly towards higher photon energy, and the Drude response (yellow line) was suppressed. Although such a blueshift is very weak, it is somewhat consistent with the more resistive V_1*−x*_W_*x*_O_2_(B) with increasing *x*. Figure 3d shows a very weak change in the electronic structure of VO_2_(B) with tungsten doping. Therefore, we suggest that the resistivity increase in V_1*−x*_W_*x*_O_2_(B) is attributable to mechanisms other than any simple change in the electronic structure.

### Mechanisms underlying the tunable resistivity of tungsten-doped VO_2_(A) and VO_2_(B)

Taking our experimental results together, we found that the resistivities of VO_2_(A) and VO_2_(M1) and those of VO_2_(B) and VO_2_(R) have similar dependences on tungsten doping. Numerous studies have attributed the stabilization of metallic V_1*−x*_W_*x*_O_2_(M1) to correlation variations, with structural distortion of V−V dimers^24–26^ and band filling by electron doping^27^, as we will explain in detail in the following paragraphs. In this stage, we aimed to understand the behaviours of V_1*−x*_W_*x*_O_2_(A) and V_1*−x*_W_*x*_O_2_(B) (*x* < *x*_c_) by adapting and modifying the mechanisms in V_1*−x*_W_*x*_O_2_(M1) and V_1*−x*_W_*x*_O_2_(R).

V_1*−x*_W_*x*_O_2_(A) shows tunable properties due mainly to correlation effects being modulated by chemical-strain-induced redistribution of V−V distances. Goodenough noted that vanadium oxides are metallic when the distance between vanadium ions is less than 2.94 Å^28^. VO_2_(M1) is insulating because correlated electrons are localized in V−V dimers, where the asymmetric distances of V−V atoms inside and between dimers are 2.65 and 3.12 Å, respectively. It is widely accepted that the transition from insulating VO_2_(M1) to metallic VO_2_(R) is accompanied by a symmetric redistribution of V−V chains, with an even distance of 2.88 Å. When vanadium ions are replaced with tungsten, the X-ray absorption fine structure indicates that the local structure around each tungsten atom is intrinsically symmetric, with a tetragonal-like structure. Therefore, the nearby V−V dimers in a VO_2_(M1) lattice are rearranged to form rutile-like VO_2_ nuclei^25,26^. In VO_2_(A), vanadium ions along the *c*-axis have alternating distances of 3.25, 3.11, and 2.77 Å at room temperature (<162 °C)^29^. Although the XRD results imply that V_1*−x*_W_*x*_O_2_(A) mostly has a tetragonal structure similar to pure VO_2_(A), expansion of the local structure due to tungsten could symmetrize the V−V chains. This rearrangement would weaken the correlation effect in V_1*−x*_W_*x*_O_2_(A), leading to lower resistivity.

**Figure 4:**
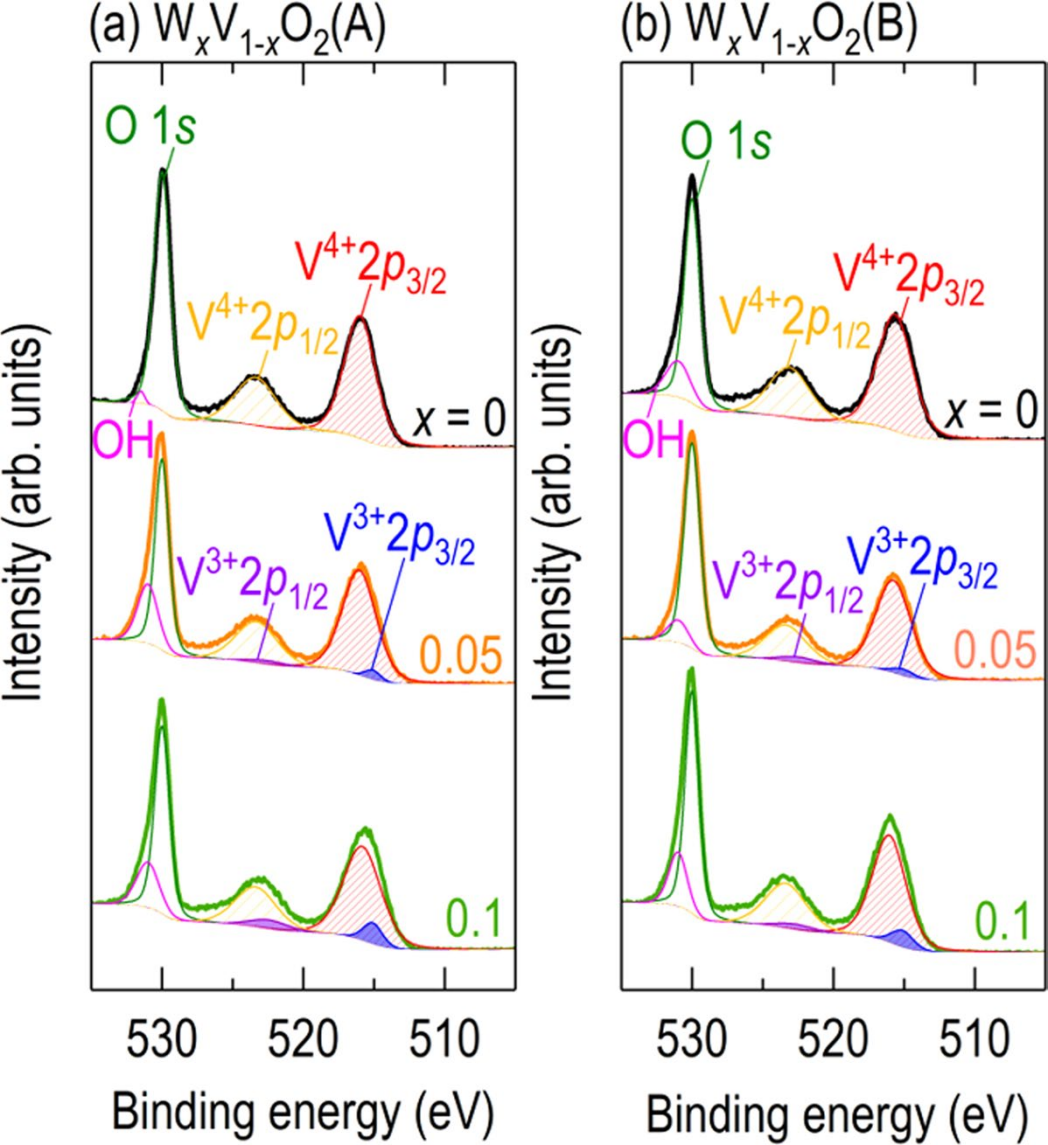
X-ray photoemission spectroscopy (XPS) V 2*p*_3/2_, V 2*p*_1/2_, and O 1 *s* spectra of (**a**) V_1*−x*_W_*x*_O_2_(A) and (**b**) V_1*−x*_W_*x*_O_2_(B) in the binding energy range of 505–535 eV. While the XPS spectra of non-doped epitaxial films are fitted by V^4+^ peaks (red and yellow patterns), V^3+^ peaks (blue and purple patterns) are additionally required for the XPS spectra of V_1*−x*_W_*x*_O_2_ (*x* = 0.05, 0.1). The intensity of the V^3+^ peaks increases with increasing *x*.

As an alternative mechanism for V_1*−x*_W_*x*_O_2_(A), we also considered electron doping since V^4+^ ions neighbouring the site of W^6+^ dopants change to V^3+^ ions to maintain charge neutrality^30^. This band filling drastically decreases the Coulomb repulsion energy and accordingly weakens the electron correlation^27^, metallizing V_1*−x*_W_*x*_O_2_(M1). We also found a significant evolution of V^3+^ oxidation states in V_1*−x*_W_*x*_O_2_(A) with tungsten doping (*x* = 0, 0.05, 0.1). Figure 4 shows XPS V 2*p*_3/2_, V 2*p*_1/2_, and O 1 *s* spectra in the binding energy range of 505–535 eV. We fitted the XPS spectra of pure VO_2_(A) (Fig. 4a) with V^4+^2*p*_3/2_ (red pattern) at 515.84 ± 0.2 eV and V^4+^2*p*_1/2_ (orange pattern) at V^4+^2*p*_3/2_ + 7.33 eV^31^, indicating that the oxidation state of our non-doped epitaxial films was firmly V^4+^. Interestingly, V^3+^ peaks [V^3+^2*p*_3/2_ (blue pattern) at 515.29 ± 0.2 eV and V^3+^2*p*_1/2_ (purple pattern) at V^3+^2*p*_3/2_ + 7.33 eV^31^] were additionally required to resolve the XPS spectra of V_1*−x*_W_*x*_O_2_(A) (*x* = 0.05, 0.1). It should be noted that the V^3+^ peaks were stronger with increasing *x*. This similar observation between V_1*−x*_W_*x*_O_2_(A) and V_1*−x*_W_*x*_O_2_(M1) indicates that electron-doping-induced band filling also plays an important role in tungsten-doped metallization^30^.

Although we also found significant evolution of the V^3+^ XPS peaks in V_1*−x*_W_*x*_O_2_(B) (Fig. 4b), it is quite interesting to note that VO_2_(B) became more insulating with increasing *x*. Therefore, we hypothesize that another mechanism, different from those for V_1*−x*_W_*x*_O_2_(A) and V_1*−x*_W_*x*_O_2_(M1), plays an important role in the tunable properties of V_1*−x*_W_*x*_O_2_(B) (*x* < *x*_c_). It should be noted that the correlation effects in VO_2_(B) and VO_2_(R) are weaker than those in VO_2_(A) and VO_2_(M1), considering their lower resistivities and narrower bandgaps^1,2^. Therefore, we suggest that the increase in resistivity in V_1*−x*_W_*x*_O_2_(B) originates from disorder-induced electron scattering. Since more dopants will scatter more electrons, the resistivity will increase with heavier doping^32,33^.

## Conclusion

Tungsten doping provided an effective way to tune the resistivity of correlated VO_2_(A) and VO_2_(B). XRD revealed that the crystal structures of V_1*−x*_W_*x*_O_2_(A) and V_1*−x*_W_*x*_O_2_(B) expanded and were well preserved for *x* < 0.1–0.15. At this low doping concentration, the resistivity of V_1*−x*_W_*x*_O_2_(A) decreased, similar to that of V_1*−x*_W_*x*_O_2_(M1), with increasing tungsten concentration; in contrast, the resistivity of V_1*−x*_W_*x*_O_2_(B) increased, similar to that of V_1*−x*_W_*x*_O_2_(R). Spectroscopic ellipsometry revealed that tungsten doping resulted in a redshift of the unoccupied *t*_2g_ orbital, the creation of an in-gap state in V_1*−x*_W_*x*_O_2_(A), and a slight blueshift of unoccupied *t*_2g_ orbitals in V_1*−x*_W_*x*_O_2_(B). Both vanadate films showed evolution of the V^3+^ oxidation states based on the XPS study. Referring to the mechanisms in correlated V_1*−x*_W_*x*_O_2_(M1), we proposed that V_1*−x*_W_*x*_O_2_(A) and V_1*−x*_W_*x*_O_2_(B) showed opposite dependences due to either chemical-strain-induced redistribution of V*−*V distances or electron-doping-induced band filling and disorder-induced electron scattering, respectively. We leave further consideration of the mechanisms for future studies.

The extreme tunability of correlated VO_2_(A) and VO_2_(B) enables their use in next-generation electronic devices, as well as energy devices. As we mentioned in the Introduction section, VO_2_(M1) has shown a reversible resistivity change due to intercalation of hydrogen^10,11^ and ionic liquid gating^12,13^. The similar doping dependence between VO_2_(A) and VO_2_(M1) suggests that these dynamic control methods would enable application of correlated VO_2_(A) [also VO_2_(B)] in memories, transistors, and gas sensors, as has been extensively studied for VO_2_(M1).

## Methods

### Epitaxial film growth of tungsten-doped VO_2_(A) and VO_2_(B)

We recently optimized the growth conditions for epitaxial films of VO_2_(A) and VO_2_(B) on either perovskite oxides or Y-stabilized ZrO_2_^2,3,16–18^. Using pulsed laser epitaxy, we deposited VO_2_(A) and VO_2_(B) epitaxial films on (011)-oriented SrTiO_3_ and (001)-oriented LaAlO_3_ substrates, respectively. We ablated a tungsten-doped V_2_O_5_ target with a KrF (248 nm wavelength) pulsed laser at a rate of 10 Hz and an intensity of 1 J cm^−2^. For the targets, we mixed WO_3_ and V_2_O_5_ powders in the desired molar ratio and sintered pellets at 650 °C for 12 hours in air. We used this low sintering temperature due to the low melting point (690 °C) of V_2_O_5_. We fixed the substrate temperature at 420 °C since VO_2_(A) and VO_2_(B) are thermodynamically unstable above 430 °C and transition into VO_2_(R) above 470 °C^2,29^. We used a flow of oxygen gas with a partial pressure, ${P}_{{{\rm{O}}}_{2}}$, of 8 mTorr for VO_2_(A) and 15 mTorr for VO_2_(B) since VO_2_ stably forms in only a narrow range of 5 mTorr <${P}_{{{\rm{O}}}_{2}}$ < 30 mTorr^16,34^.

### Measurement of electrical and optical properties

To investigate the electrical transport properties, we used a physical property measurement system (Quantum Design Inc.). We used the four-point probe method, which is the most common method for measuring the resistivity^35^. We deposited evenly spaced Pt contacts on the middle of the film surface. We applied a small constant current through the outer two contacts and measured the voltage between the inner two contacts. We swept the temperature in the range of 10–400 K. We measured the reflectance, *R*(*ω*), spectra in a photon energy range of 0.1–1 eV via a Fourier transform-type infrared spectrometer (model VERTEX 70 v; Bruker). We employed an *in situ* gold overcoating technique to obtain an accurate absolute value of *R*(*ω*). We obtained the optical conductivity of the VO_2_ film via a two-layer model fit of the measured *R*(*ω*) with Drude-Lorentz oscillators^23,36^. We used spectroscopic ellipsometers (models V-VASE and M-2000; J. A. Woollam Co.) to obtain the complex dielectric constants, $\epsilon (\omega )={\epsilon }_{1}(\omega )+i{\epsilon }_{2}(\omega )$, in the energy region between 1 and 5 eV. The optical conductivity, *σ*_1_(*ω*), in this energy range can be calculated by ${\sigma }_{1}(\omega )={\epsilon }_{0}\omega {\epsilon }_{2}(\omega )$^23^, where ${\epsilon }_{0}$ is the vacuum permittivity.

### Characterization of structural properties and oxidation states

We investigated the crystal structures via a four-circle high-resolution X-ray diffractometer (model Empyrean; PANalytical) using Cu radiation with a wavelength of 1.5406 Å. Using the fringe patterns obtained in X-ray reflectivity measurements, we confirmed that the films had an ~100-nm thickness (Fig. S2). To determine the oxidation states of vanadium, we carried out XPS (model ESCALAB 250Xi; Thermo Scientific) using a monochromatic Al source with a photon energy of 1486.6 eV under an environmental pressure of 10^−8^ Torr. To remove contamination, the film surface was sputtered with argon ions for 10 seconds^16^. We used the O 1 *s* peak at 530.0 eV as the energy reference. We supplied electrons using an electron gun to avoid any charging effect.

## Supplementary information

Supplementary information.
