# Supplementary material for: Tunable resistivity of correlated VO2(A) and VO2(B) via tungsten doping
Source: Sci Rep. 2020 Jun 16;10:9721. doi: 10.1038/s41598-020-66439-2 (PMC7297976; doi:10.1038/s41598-020-66439-2)
Supplement: Supplementary file 1 — Supplementary information. [file 41598_2020_66439_MOESM1_ESM.docx]

Supplemental material

**Tunable resistivity of correlated VO_2_(A) and VO_2_(B) via tungsten doping**

Songhee Choi^1^, Gihyeon Ahn^2^, Soon Jae Moon^2^, and Shinbuhm Lee^1,^*

^1^Department of Emerging Materials Science, Daegu-Gyeongbuk Institute of Science and Technology, Daegu 42988, Republic of Korea
^2^Department of Physics, Hanyang University, Seoul 04763, Republic of Korea

*E-mail: lee.shinbuhm@dgist.ac.kr

**1. Structural properties of tungsten-doped VO_2_(A) and VO_2_(B)**

We grew V_1_*_−x_*W*_x_*O_2_(A) and V_1_*_−x_*W*_x_*O_2_(B) epitaxial films on (011)SrTiO_3_ and (001)LaAlO_3_, respectively, for *x* from 0 to 0.25. As shown by the X-ray diffraction (XRD) *θ*−2*θ* scans in Figure S1a, we found two strong peaks near 2*θ* = 42.4° and 65.7°, as well as strong (011) and (022)SrTiO_3_ peaks at 2*θ* = 32.4° and 67.6°. These peaks appear due to diffraction from the (400) and (600) planes of VO_2_(A) in reference to powder diffraction. The V_1_*_−x_*W*_x_*O_2_(A) epitaxial films are (100)-oriented. The XRD *θ*−2*θ* scans in Figure S1b indicate that the V_1_*_−x_*W*_x_*O_2_(B) films are (001)-oriented since there are strong (001), (002), and (003)VO_2_(B) diffraction peaks near 2*θ* = 14.4°, 28.9°, and 44.1°, as well as strong (001) and (002)LaAlO_3_ peaks at 2*θ* = 23.5° and 47.9°. In our previous report^1^, we provided a full explanation of the epitaxial relationships between VO_2_(A) and (011) perovskite oxides and between VO_2_(B) and (001) perovskite oxides.


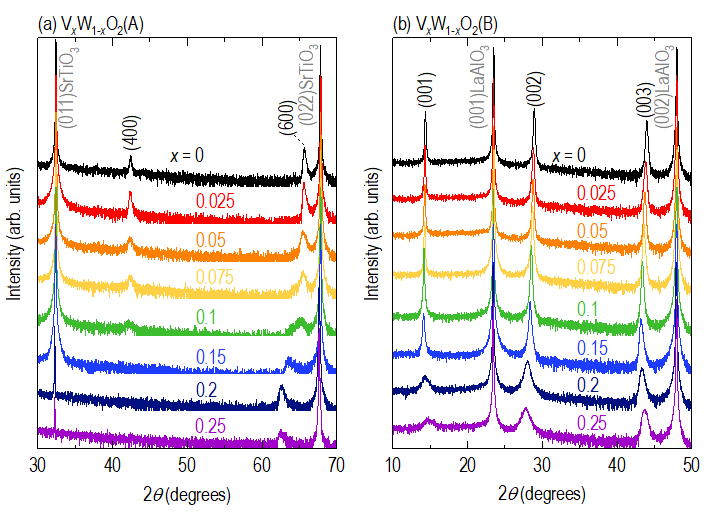


**Figure S1.** X-ray diffraction (XRD) *θ*‒2*θ* scans for (a) V_1_*_−x_*W*_x_*O_2_(A) (*x* ≤ 0.25) epitaxial films grown on (011)-oriented SrTiO_3_ and (b) V_1_*_−x_*W*_x_*O_2_(B) epitaxial films grown on (001)-oriented LaAlO_3_. The diffraction peaks are preserved for *x* < *x*_c_ ≈ 0.1–0.15, but the intensities become weaker for *x* > *x*_c_ due to film deterioration. With increasing tungsten concentration, V_1_*_−x_*W*_x_*O_2_(A) and V_1_*_−x_*W*_x_*O_2_(B) show a shift in their diffraction peaks towards lower 2*θ* values.

**2. Film thicknesses of tungsten-doped VO_2_(A) and VO_2_(B)**

To check the film thickness, we used X-ray reflectivity (XRR). Figure S2 shows clear fringe patterns for V_1_*_−x_*W*_x_*O_2_(A) and V_1_*_−x_*W*_x_*O_2_(B) epitaxial films. Using the fringe patterns, we confirmed the thicknesses of ~100 nm. These XRR fringe patterns also highlight that all the films used in this work have a commensurate interface and a flat film surface. We failed to obtain fringe patterns for V_0.75_W_0.25_O_2_(A) and V_0.75_W_0.25_O_2_(B), probably due to the increase in the surface roughness.

**Figure S2.** X-ray reflectivity results of (a) V_1_*_−x_*W*_x_*O_2_(A) and (b) V_1_*_−x_*W*_x_*O_2_(B) epitaxial films.

**3. Indirect and direct bandgaps in tungsten-doped VO_2_(A) and VO_2_(B)**

We estimated the bandgap by calculating the absorption coefficient $\alpha=\frac{2\kappa\omega}{c}$, where *κ* is the extinction coefficient, *c* is the speed of light in free space, and *ω* is the angular frequency of light^2^. As shown in Figure S3a, we obtained an indirect bandgap of ~0.9 eV for VO_2_(A) by plotting $\alpha^{\frac{1}{2}}\propto(\hbar\omega-E_{g})$, where $\hbar\omega$ is the photon energy and *E_g_* is the optical bandgap^2^. We estimated the bandgap by linearly extrapolating the absorption edge to zero, as exemplified by the dashed lines. The indirect bandgaps of V_1_*_−x_*W*_x_*O_2_(A) (*x* ≠ 0) and V_1_*_−x_*W*_x_*O_2_(B) are negligible (Figures S3a and b). For reference, we also obtained the direct bandgaps by plotting $\alpha^{2}\propto(\hbar\omega-E_{g})$ (Figures S4a and b).


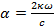

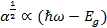

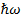

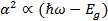


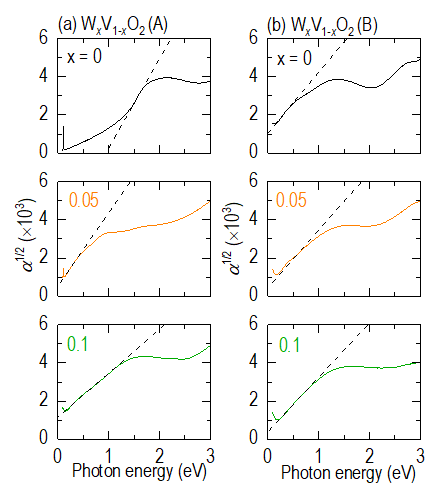


**Figure S3.** Absorption coefficient as a function of photon energy, used to estimate the indirect bandgaps of (a) V_1_*_−x_*W*_x_*O_2_(A) and (b) V_1_*_−x_*W*_x_*O_2_(B) epitaxial films. To determine the bandgaps, we extrapolated the linear portion of the sharp rise of the absorption coefficient curve to zero, as indicated by the dashed lines.


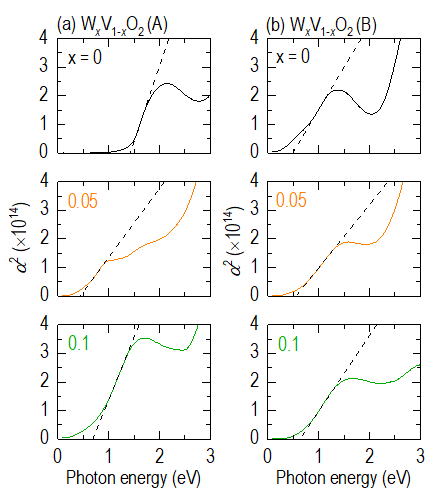


**Figure S4.** Absorption coefficient as a function of photon energy, used to estimate the direct bandgaps of (a) V_1_*_−x_*W*_x_*O_2_(A) and (b) V_1_*_−x_*W*_x_*O_2_(B) epitaxial films. The dashed lines were used for the same purpose as those in Figure S3.

**References**

[1] Lee, S., Ivanov, I. N., Keum, J. K. & Lee, H. N. Epitaxial stabilization and phase

instability of VO_2_ polymorphs. *Sci*. *Rep*. **6,** 19621 (2016).

[2] Fox, M. *Optical properties of solids.* (Oxford University Press, Oxford, United Kingdom, 2010).
